# Factors that influence regional resilience planning in Central Karoo, South Africa

**DOI:** 10.4102/jamba.v14i1.1265

**Published:** 2022-04-28

**Authors:** Elizabet Dimitrova, Masilonyane Mokhele

**Affiliations:** Department of Urban and Regional Planning, Faculty of Informatics and Design, Cape Peninsula University of Technology, Cape Town, South Africa

**Keywords:** regional resilience, Central Karoo, South Africa, resilience planning, Western Cape, Beaufort West, Prince Albert, Laingsburg

## Abstract

Applied in various disciplines, the concept of resilience has become a catchword in academic and policy discourse across the world. Despite the rapidly growing interest, there is a dearth of literature on resilience in the context of rural areas. To contribute towards extending the existing knowledge, this article analyses factors that influenced the low levels of incorporation of regional resilience in the plans (spatial development frameworks [SDFs]) that guide planning and development in a rural region of Central Karoo, South Africa. The study that the article draws upon revolved around qualitative interviews conducted with seven key informants who were knowledgeable about social, economic and environmental challenges as well as planning and development in the Central Karoo region. The semi-structured interviews were conducted through the online platform of Microsoft Teams. Against the background of low levels of incorporation of regional resilience in the plans that have a bearing on planning and development in the Central Karoo region, it was discovered that the lack of knowledge, financial and human resource constraints and a lack of synergy between different stakeholders were the main reasons for the inadequate incorporation of regional resilience in the subject SDFs. To improve this state of affairs, it is recommended that the stakeholders in the region be empowered on matters pertaining to regional resilience. In terms of empirical research, it is recommended that future studies go beyond the analysis of the content of plans in the manner of this article and analyse the actual regional resilience of rural areas.

## Introduction

Reminiscent of the so-called fuzzy concepts (Markusen 1999), resilience is increasingly becoming a catchword, which fosters enthusiasm among academics and policymakers across the globe (Eraydin & Tasan-Kok 2013a; Stead & Tasan-Kok 2013). The literature widely acknowledges that resilience is derived from the Latin word *resilire*, which denotes to spring back (Modica & Reggiani 2015). Despite its newly discovered and rapidly growing fame, resilience is not a recently coined word. In the context of physics, resilience was first defined in 1824 as the potential of a material to return to its original form (i.e. in terms of size and shape) following distortion due to a compressive shock (Modica & Reggiani 2015). Resilience was subsequently adopted by numerous disciplines (Modica & Reggiani 2015) including the social sciences in general, where it is typically used as a framework for interrogating complex social systems. Three overarching facets of resilience can be identified in the literature, namely engineering, ecological and evolutionary resilience. These facets accordingly inform a multitude of definitions of resilience, which vary between and among different disciplines and fields of study.

Engineering resilience is concerned with the ability of a system to revert to its equilibrium position following the occurrence of a disturbance or a shock, the effectiveness of which is ascertained by the speed at which that system is able to return to its previous stable state (Boschma 2015; Carpenter, Westley & Turner 2005; Holling 1996; Vale 2014). In dissecting the facet of ecological resilience, contemporary literature largely draws on the seminal work of Holling (1973, 1996), whose definition hinges on the level of disturbance that can be sustained by a given system. Holling (1973) argues that because an equilibrium-based approach (in the manner of engineering resilience) is intrinsically static, it fails to provide insight into the transient behaviour of systems that do not get close to a state of equilibrium. Ecological resilience is thus concerned with the ability of a system to absorb change, adapt accordingly and carry on (Adger 2000; Ahern 2011; Davoudi 2012; Holling 1973) as opposed to reverting to the previous state of equilibrium. In the context of urban and regional systems, it can be argued that a state of equilibrium does not exist and is neither feasible nor desirable due to, among others, complex social dynamics at play (e.g. see Bailey 1984). This implies that systems do not stand still or revert to a stable condition following the occurrence of disturbances or shocks (Turok 2014). It is therefore important to overview the third facet of resilience, which various scholars (for instance, eds. Berkes & Folke 1998; Carpenter et al. 2005; Folke 2006; Scheffer 2009) refer to as evolutionary resilience, adaptive resilience, socio-ecological resilience or bounce-forward. Although it is acknowledged that external factors can affect a given system (Turok 2014), the evolutionary way of thinking about resilience is based on the argument that systems themselves may change without the occurrence of disturbances or shocks; hence resilience cannot be simply conceived as a return to a previous state of normality (Elmqvist, Barnett & Wilkinson, 2013).

Against the background of the interrelated facets of resilience, as reflected by among others the extensive literature review by Meerow, Newell and Stults (2016), there is an abundance of research focusing on the resilience of towns, cities and metropolitan or urban regions. This dominant focus is encapsulated in the notion of urban resilience. There is however a relative paucity of literature on resilience in the context of rural areas (for exception, see Kapucu, Hawkins & Rivera 2013; Melore & Nel 2020). It can be argued that rural areas have specific characteristics and somehow peculiar challenges, which cannot be appropriately addressed through generalised regional policy (Van Aswegen & Retief 2020) that is largely informed by the circumstances of urban areas. Relatedly, resilience (or resilience planning in the context of the study) must be analysed and interpreted in relation to uneven spatial development across a range of geographical scales (Smith [1990] cited in Mackinnon & Derickson 2012) wherein urban areas are typically prioritised over rural areas in the manner of the explanation advanced by the theory of urban bias (Lipton 1977). In this regard, resilience planning should be approached differently in rural areas to appropriately respond to the specific context and circumstances of rurality (Fox-Lent & Linkov 2018).

The said gap in the resilience-related literature (i.e. inadequate focus on rural areas) is against a backdrop of the argument that academics and policymakers have not given sufficient attention to the integration of resilience planning with spatial or development planning (Eraydin & Tasan-Kok 2013b). To contribute towards filling this gap, the broad aim of this study is to analyse regional resilience planning in the context of rural areas of South Africa. Similar to the notion of resilience in general, one of the reasons for the wide currency of the term regional resilience in the contemporary literature and policy debates is arguably its fuzziness, where it can mean different things to different people at different times. In the social sciences, regional resilience has particularly become popular because of its association with the notion of regional adaptation. Regional adaptation attempts to address the question of why some regions manage to overcome (economic) distress and maintain a high quality of life for communities while other regions fail to overcome adversity (Christopherson, Michie & Tyler 2010).

Towards addressing the aim of the research above, this article uses the study area of a rural region of Central Karoo in South Africa to analyse factors that influence the incorporation of regional resilience in the plans (i.e. spatial development frameworks [SDFs]) that guide planning and development in the region. This study attempts to explain the findings of a related study (Dimitrova 2021) in which it was discovered that the SDFs in the Central Karoo region did not adequately acknowledge and incorporate the phenomenon of regional resilience. In South Africa, SDF is a core component of integrated development planning, which should be aligned with, inter alia, disaster management plans, financial plan, development priorities, and operational strategies of a municipality (Republic of South Africa 2000). Notwithstanding the debates on their efficacy in guiding development (Todes 2008), SDFs arguably have the potential to generate resilience if they are comprehensively formulated and implemented accordingly.

In evaluating the incorporation of regional resilience in the SDFs, the aforementioned study used content analysis to examine the extent of acknowledgement of the environmental, social and economic challenges in the Central Karoo region as well as the normative position advanced towards resolving the existing problems or circumventing potential challenges and disasters in the region. In other words, a balanced combination of the two aspects (i.e. challenges and potential solutions) was used as a proxy for the acknowledgement of regional resilience in the SDFs analysed.

## Research methods

### Study area

**FIGURE 1:**
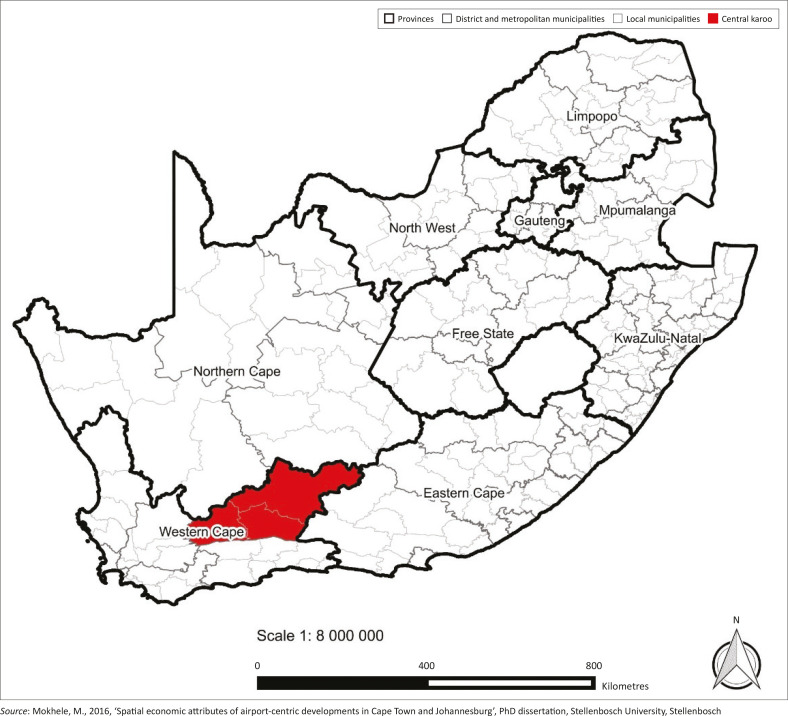
Location of Central Karoo region.

As alluded to in the introduction, the article is based on the study area of Central Karoo region, which is located in the Western Cape province of South Africa. As a reflection of the diversity of the region, Central Karoo is bounded by two provinces, namely Northern Cape province to the north and Eastern Cape province to the east, including numerous neighbouring municipalities (Figure 1). Aligned with the administrative boundaries of the Central Karoo district municipality, the region comprises three local municipalities, namely, Beaufort West, Laingsburg and Prince Albert. Although the region covers an extensive land area of about 39 000 km^2^ (which is approximately 28% of the province’s total land area), it has a minute population of 74 247 people (Statistics South Africa 2018).

Central Karoo region was considered a suitable study area towards addressing the research aim because of three primary reasons. Firstly, the region is a predominantly rural area (Western Cape Government 2017), which is situated on the periphery of the Western Cape province, geographically far from the City of Cape Town metropolitan area and the surrounding functional region. Secondly, the climate of the region is dry and the area is consistently subjected to extreme weather conditions, particularly droughts and heat waves. Thirdly, Central Karoo region has large deposits of shale gas. This unique natural advantage has brought to the table proposals on fracking or hydraulic fracturing, which would be put in place to extract the valuable gas from the earth’s surface. Some sections of society (including scientists and environmental activists) are sceptical about the proposals and argue that fracking would bring detrimental health risks to the populace (e.g. Willems 2015) as well as a multitude of hazards to the region’s precious natural environment (e.g. eds. Scholes et al. 2016). These three attributes arguably raise concerns on the vulnerability and resilience of the region, and therefore made the Central Karoo apt for the investigations of resilience planning in a rural context. The notion of vulnerability denotes the degree to which a system is susceptible to harm albeit it could be argued that vulnerability is not necessarily dependent on the probability of the occurrence of a shock (Smit et al. 1999).

### Data collection

The study that the article draws upon was based on qualitative interviews conducted with seven key informants who were knowledgeable about planning and development in the Central Karoo region (Table 1). The informants were specifically selected for participation in the study because they had first-hand experience with the community challenges and/or the drafting and implementation of SDFs in the Central Karoo region. These are Central Karoo district SDF, Beaufort West municipal SDF, Laingsburg municipal SDF and Prince Albert municipal SDF. The informants occupied leadership positions in the respective organisations; hence it was deemed they would have comprehensive and up-to-date knowledge about the region. They were from different spheres of government (i.e. provincial government, district municipality and the constituent local municipalities) as well as the non-governmental organisation (NGO) sector to, at least in part, provide the perspective of the community.

**TABLE 1:** Key informants interviewed.

Respondent number	Organisation	Reason for selection	Date of interview
Respondent 1	Provincial Government Western Cape: Department of Environmental Affairs and Development Planning	Research and compilation of the Central Karoo District SDF	19 July 2021
Respondent 2	Central Karoo district municipality	Familiarity and involvement with the compilation of the Central Karoo SDF	26 July 2021
Respondent 3	Laingsburg local municipality	Implementation of the local SDF	19 July 2021
Respondent 4	Prince Albert local municipality	Familiarity and involvement with the compilation of the local SDF	26 July 2021
Respondent 5	Beaufort West local municipality	Implementation of the local SDF	26 July 2021
Respondent 6	Beaufort West local municipality	Familiarity and involvement with the compilation of the local SDF	19 July 2021
Respondent 7	UCT Rural Support Network	Knowledge of social and economic issues in the region	07 October 2021

SDF, spatial development frameworks.

The identification of the key informants was based on the non-probability sampling technique of snowballing wherein the number of the respondents did not have to be representative or statistically significant in the manner of quantitative research. The snowball process began with the identification of a few individuals, and spread out on the basis of the links, recommendations and referrals made by the individuals contacted initially (Gobo 2004). This implies that the respondents were not randomly selected and would have, in one way or another, been professionally connected (Neuman 2000) or were even acquaintances. It should also be noted that due to ethics-related reasons, it was decided that the names and positions of the respondents not be disclosed in the article, but rather reference only be made to their respective organisations.

The semi-structured interviews were conducted (in July and October 2021) virtually via the Microsoft Teams online platform, where each interview lasted between 20 and 30 min. The interviews were guided by a semi-structured questionnaire (Table 2), which entailed a combination of questions, namely, closed-ended, open-ended and Likert scale. With regard to open-ended questions, the respondents were given an opportunity to provide any appropriate answer, as opposed to restricting the answers to predetermined options in the manner of closed-ended questions. The open-ended questions were particularly important in the investigations, and their significance towards addressing the objective of the study is discussed further in the imminent section on data analysis.

**TABLE 2:** Interview guide.

Number	Question
1.	What is your level of understanding of the concept of resilience?
2.	Is resilience well catered for in the Central Karoo District SDF?
3.	Is resilience well catered for in the local SDF(s)?
4.	What influences the poor incorporation of resilience in the SDFs?
5.	In your view, what are the main environmental challenges experienced in the area?
6.	Are those environmental challenges adequately addressed in the Central Karoo District SDF?
7.	Are those environmental challenges adequately addressed in the local SDF(s)?
8.	What influences the poor attention to environmental challenges?
9.	In your view, what are the social challenges experienced in the area?
10.	Are those social challenges adequately addressed in the Central Karoo District SDF?
11.	Are those social challenges adequately addressed in the local SDF(s)?
12.	What influences the poor attention to social challenges?
13.	In your view, what are the economic challenges experienced in the area?
14.	Are those economic challenges adequately addressed in the Central Karoo District SDF?
15.	Are those economic challenges adequately addressed in the local SDF(s)?
16.	What influences the poor attention to economic challenges?

*Source*: Adapted from Dimitrova, E., 2021, ‘Planning for regional resilience in Central Karoo, Western Cape, South Africa’, Master’s thesis, Cape Peninsula University of Technology, Cape Town

SDF, spatial development frameworks.

In the light of coronavirus disease 2019 (COVID-19) pandemic and the resultant lockdown restrictions, it was not ideal to meet and interview the respondents in person, that is, face-to-face. However, this decision did not impact negatively on this study. The literature has shown that the benefits of telephone interviews include that they are flexible and they almost match the advantages of face-to-face interviews albeit their duration should ideally be shorter for the purposes of maintaining rapport. Furthermore, in comparison to face-to-face interviews, telephone interviews are relatively cheaper and can be executed quickly (Block & Erskine 2012; Neuman 2000) particularly because no travelling is required. In this regard, the online platform of Microsoft Teams used in the study was regarded as an advanced form of telephonic interviewing, which comprises voice and video capabilities.

### Data analysis

To put the discussion on the analysis methods and techniques in perspective, it should be noted that the strategy for evaluating the incorporation of regional resilience in the SDF documents (i.e. in the related study mentioned in the introduction) hinged on the existing and potential environmental, social and economic problems in the region. The logic was that the acknowledgement of and response to these context-specific problems would be representative of regional resilience regardless of whether the SDF documents contained the word resilience. This underlying logic meant that the study had to seek the respondents’ views on reasons the environmental, social and economic problems were not adequately addressed in the SDFs (refer to Table 2). These views were then regarded as representative of reasons for the inadequate incorporation of regional resilience in the SDFs. Due to the vagueness of the word resilience, a decision was taken not to ask the question specifically on regional resilience because the understanding would differ among the respondents as well as between the respondents and the researcher. It was rather decided to break down regional resilience into more neutral components to ensure that the researcher and the respondents were discussing the same phenomenon.

Against this backdrop, the data collected from the qualitative interviews conducted with the seven key informants was analysed in the computer programme of Atlas.ti (version 9). There are numerous software packages that can be utilised in qualitative research, including Atlas.ti, MxQDA and NVivo (Richards 2009). Atlas.ti was selected for use in the study because the researchers’ institution had a licence to the software. Although it is ideal to thoroughly compare and systematically choose one package, Richards (2009) acknowledges that the availability of a software in an institution may influence the choice. Nonetheless, some scholars herald Atlas.ti as one of the most popular qualitative data analysis software (Lewis 2004).

Because some of the respondents did not agree to the interviews being recorded on the Microsoft Teams platform, it was decided that the best approach would be to capture the responses (on the Microsoft Excel spreadsheet) during the interviews and compose notes immediately afterwards (Rapley 2004). This decision ensured consistency in the approach to the analysis as opposed to analysing recorded interviews (i.e. voice) for some respondents and not for others who did not consent to the recording. The notes drafted from the seven interviews were inputted into Atlas.ti for analysis. The analysis specifically relied on open-ended questions (relating to the reasons for the poor incorporation of regional resilience in the SDF documents), which were used to generate word clouds based on the answers provided by the respondents. The word clouds graphically illustrated the prevalence and magnitude of the words mentioned by the respondents.

### Ethical considerations

Approval to conduct the study was obtained from the Faculty of Informatics and Design Research Ethics Committee at Cape Peninsula University of Technology, reference number: 211006513/2020/5.

## Findings on factors that influence the incorporation of regional resilience in the spatial development frameworks

Based on the data collection and analysis methods outlined above, the current section presents the findings on the factors that influenced the level of incorporation of regional resilience in the plans (SDFs) that have a bearing on planning and development in the Central Karoo region. As noted in the introduction, the analysis was against the background of the findings that discovered low levels of incorporation of regional resilience in the subject SDFs wherein the environmental, economic and social challenges and the associated normative stands were used as a proxy for the acknowledgement of regional resilience. The presentation below is therefore structured around the environmental, economic and social aspects, which are subsequently synthesised to draw the commonalities and differences in the factors uncovered.

### Environmental matters

**FIGURE 2:**
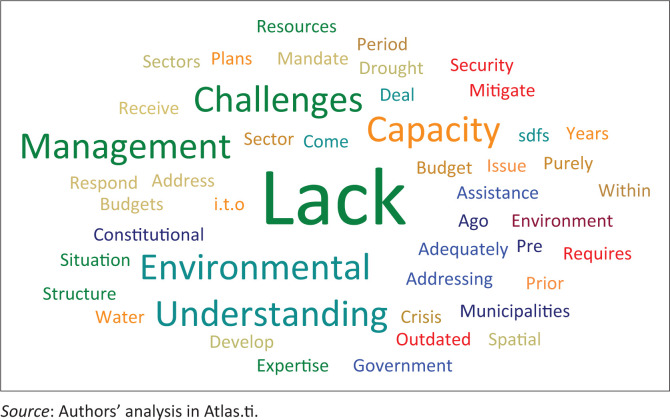
Reasons for poor attention to environmental issues in the spatial development frameworks.

The word cloud of the factors advanced by the respondents on the poor acknowledgement of environmental issues in the subject SDFs is graphically depicted in Figure 2. ‘Lack’ was the frequently mentioned word, which is elaborated on in the narrative hereunder in relation to other expressions in the word cloud.

The dire lack of resources (financial and human) and the associated lack of capacity were highlighted as the main reasons why the environmental challenges in the Central Karoo district and the constituent local municipalities were not adequately addressed in the SDFs. A particularly stressed point was the lack of funding at the level of district municipality and the constituent local municipalities. One of the directors at Beaufort West local municipality emphasised the detrimental impact of the lack of assistance from other spheres of government in the efforts to mitigate the ramifications of the drought in the region. Although it was not clear how such assistance would have directly resolved the problems, the area would have in one way or another benefited from the additional resources and support from other levels of government (Respondent 5). The problem of insufficient resources was particularly mentioned by the managers of municipalities across the region, who pointed out that the local municipalities were understaffed and as a result heavily constrained in undertaking the requisite planning (and implementation) tasks. In addition, a town and regional planner from the Western Cape provincial government stressed that for problems like water scarcity in the region, there was a need for a number of sectors to respond and participate towards resolving the problems. The respondent argued that the water problem was not a purely spatial planning issue; hence it could not be adequately addressed within the SDFs in the region without the participation of other stakeholders (Respondent 1). A poor understanding of the role of local municipalities in environmental management was stressed by the manager of strategic services at the Central Karoo district municipality. This constraint negatively affected the effectiveness of environmental strategies formulated at the higher levels of government, particularly how they could be adapted to local conditions and implemented accordingly (Respondent 2).

The majority of the respondents pointed out that the lack of a clear, implementation focus pertaining to the environmental management plan was one of the key reasons why the environmental challenges were not given sufficient attention in the SDFs and the associated strategies and programmes. This gap showed that there was a dire need for more focused environmental management planning towards crafting a regional strategy, which would then inform initiatives at the local level. A senior planner at Beaufort West local municipality asserted that the requisite budgets were either not made available or they were very limited for the purpose of developing sector plans to effectively address environmental management in the region. Although critical, it was noted that the implementation aspect was often overlooked by the municipalities. This situation left the officials and other stakeholders in the region with a poor understanding on how to compile the environmental plans and ultimately implement them (Respondent 6).

### Social matters

**FIGURE 3:**
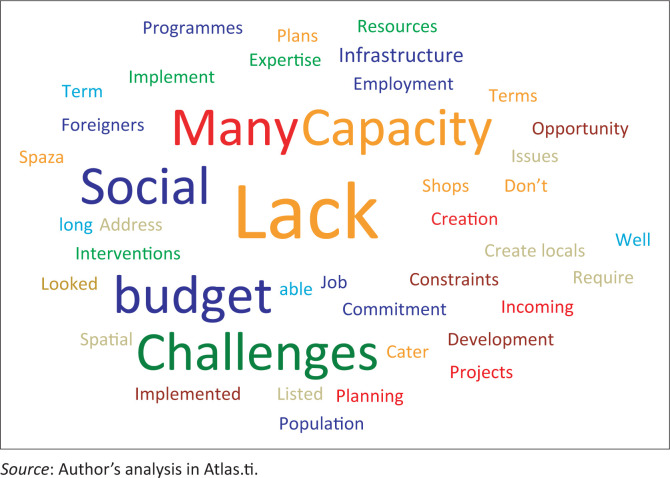
Reasons for poor attention to social issues in the spatial development frameworks.

Figure 3 depicts the word cloud of the factors advanced by the respondents on the poor acknowledgement of social issues in the subject SDFs in the Central Karoo region. Similar to the foregoing discussion, ‘lack’ is notably by far the dominant expression in the word cloud, which is discussed hereunder relative to other words in the cloud.

The respondents asserted that social challenges in the region could be approached through many lenses or perspectives and potentially be addressed through numerous strategies in the SDFs. The provincial government’s planner stressed that the SDFs cannot alone address the social challenges of any region or area. It was noted by the respondent that many social challenges do not necessarily require spatial interventions (as contained in the SDFs) and would possibly be linked to economic interventions. These issues therefore needed to be looked at through a multi-sectoral perspective and through solutions formulated at various levels of government (Respondent 1). This assertion that the SDFs cannot be used to address many social challenges is however an interesting observation by the respondent because municipal SDFs are expected to integrate different sectors that are relevant to planning and development. This integrative function is for instance explicitly expressed in Section 21 of the *Spatial Planning and Land Use Management Act* (Republic of South Africa 2013). Other respondents noted that although there were policies aimed at addressing some of the social challenges in the region (like substance abuse, crime and violence), the lack of well-considered strategies and programmes remained one of the main stumbling blocks to addressing the challenges in the region (Respondent 2).

Beaufort West municipal officials pointed out that with regard to social issues in the region (like with the economic and environmental problems), the inadequate allocation of skills and resources was a continuing limitation that negatively impacted planning processes. This scarcity affected the Central Karoo region in general, and looking specifically into the three constituent local municipal areas, the scarcity of resources became worse. This sentiment was shared by the key informants from Laingsburg and Prince Albert local municipalities wherein it was noted that there was a lack of social programmes, which emanated from the lack of financial and human resources in the municipalities. Such interventions and programmes require a large amount of investment and skilled workforce, such as teachers, librarians, psychologists and adult educators. These programmes have to be factored into the long-term development plans and strategies in order to support the growing population, resolve the existing social issues and accordingly circumvent potential problems in the region.

### Economic matters

**FIGURE 4:**
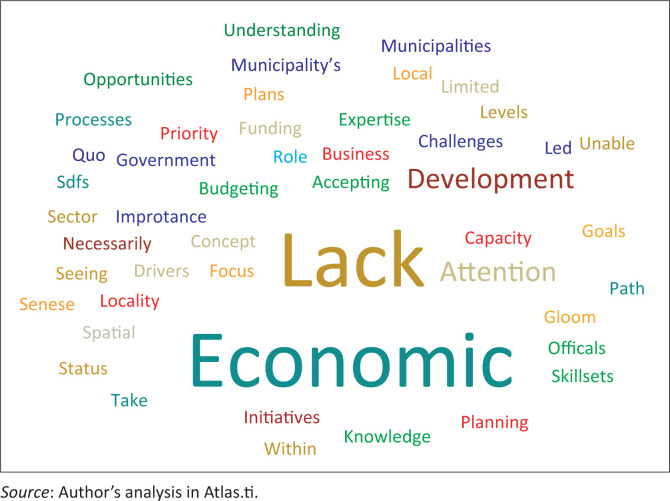
Reasons for poor attention to economic issues in the spatial development frameworks.

The word cloud of the factors advanced by the respondents on the poor acknowledgement of the economic issues in the subject SDFs is depicted in Figure 4. Similar to the foregoing discussion on the environmental and social issues, the expression ‘lack’ is considerably the most dominant in the word cloud. This is discussed hereunder in connection to other expressions in the word cloud.

A respondent from Beaufort West local municipality stressed that economic challenges in the region were not well addressed in the SDFs mainly because of the lack of resources and investment from other spheres of government (Respondent 5). This sentiment was also evident in the responses given by the Western Cape provincial chief town planner, who pointed to the inadequate resource allocation in the region. The respondents also mentioned the ramifications of the perceived acceptance that the current bad economic situation in the region would not improve. This pessimistic standpoint in part influenced the officials and drafters of the SDFs to (inadvertently) give poor attention to the economic problems in the region. In this way, the acceptance of the dire economic situation as a norm discouraged officials to be creative and advance new economic strategies in the SDFs. This included accepting that no significant investment would be made into the area because of its rural character and the perceived low economic value (Respondent 1) and/or potential. Similarly, the municipal manager of Prince Albert local municipality stated that the lack of resources was a key impediment to effectively planning for and addressing economic challenges in the region. The resources of the local municipalities were too stretched into addressing basic day-to-day service delivery to be able to plan for, among others, skills enhancement, which would in part contribute to resolving the economic problems in the region (Respondent No. 4).

Beaufort West municipality’s senior planner asserted that the main reasons for the inadequate focus on the economic challenges included the fact that the area had no defined local economic development (LED) aspirations. There was also a lack of focus on strategies pertaining to economic development, as well as a lack of significant economic drivers in the Central Karoo region. The respondents described the economies of towns and villages in the region to be on the verge of collapsing, with only a few small businesses being sustained and no new business opportunities created. This state of affairs in part pointed to the lack of planning and inadequate support provided by the public sector, including upskilling, funding as well as other development and investment opportunities. In turn, private investment could not be attracted to the region due to the lack of business opportunities. It was therefore vitally important that local government prioritise LED and come up with more plans and strategies that would attract investment for small businesses in the region (Respondent 6). A similar concern was expressed by the Central Karoo district municipality’s manager of strategic services, who noted that the officials of local municipalities in the region did not understand LED and the support that was required to address this pertinent matter (Respondent 2). This lack of understanding implied that the critical matter of LED would not be adequately addressed in the SDFs.

## Discussion and synthesis

**FIGURE 5:**
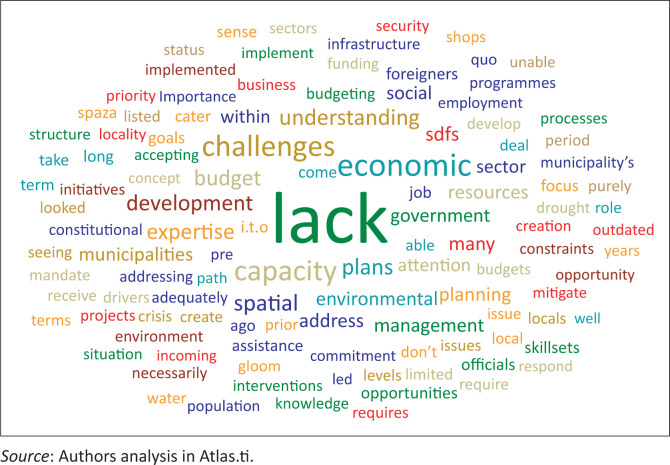
Reasons for poor attention to environmental, economic and social issues in the spatial development frameworks.

Figure 5 depicts a synthesised word cloud of the factors that influenced the poor incorporation of regional resilience in the Central Karoo district SDF and the constituent local municipal SDFs of Beaufort West, Laingsburg and Prince Albert. This word cloud is a composite of the responses advanced by the key informants on environmental, economic and social issues (as proxies for regional resilience) in the SDFs.

Similar to the foregoing separate discussions on environmental, economic and social aspects, ‘lack’ is the most frequently mentioned expression in the consolidated word cloud. This can be combined with other expressions to denote lack of understanding of regional resilience; lack of capacity, expertise and drive towards addressing the challenges of the region; lack of financial and human resources required for planning and implementation efforts; lack of commitment by the various stakeholders; and lack of integration between the different sectors and spheres of governance towards resolving the existing problems in the region as well as circumventing potential challenges and disasters.

The findings of the study support the existing literature on the dire performance of the majority of municipalities in South Africa. For instance, a number of scholars argue that the factors that influence the failure of municipalities in the country include inadequate human resources, capacity problems, shortage of skills (e.g. Koma 2010; Ramutsheli & Van Rensburg 2015) as well as the poor alignment and integration between the different spheres of government (Kanyane 2014). Van Aswegen (2018) concurs that the success of regional policy is largely dependent upon the cooperation of different spheres of government, showing that the lack of meaningful collaboration between stakeholders is a stumbling block to appropriate regional resilience planning in the Central Karoo region.

## Conclusion

The article analysed the factors that resulted in the low level of incorporation of regional resilience in the SDFs that guide planning and development in the rural region of Central Karoo, in the Western Cape province of South Africa. A non-probability sampling technique of snowballing was used to identify seven key informants or respondents who were knowledgeable of the multifaceted challenges of the region as well as planning and development in general. During the interviews (which were conducted virtually via the Microsoft Teams platform), the responses were captured on the Excel spreadsheet of a semi-structured questionnaire used as the interview guide. The responses to open-ended questions were subsequently expanded into notes for analysis in the computer programme of Atlas.ti, from which word clouds were generated to reflect the importance and magnitude of the words mentioned by the respondents. With the word ‘lack’ being the dominant expression in the resultant word clouds, it was discovered that the lack of knowledge of matters that pertain to regional resilience, resource (financial and human) constraints and the lack of integration of different spheres of governance were the main reasons for the poor level of incorporation of regional resilience in the subject SDFs (i.e. Central Karoo district SDF, Laingsburg SDF, Prince Albert SDF and Beaufort West SDF). The findings show that environmental, economic and social problems in the region are likely to continue if the SDFs are not comprehensively formulated and implemented. These challenges include the ramifications of climate change, drought, and fracking, amid the possible population growth in the region.

To improve regional resilience planning in rural regions, it is proposed that the different levels of governance augment the training, skills transfer and empowerment of government officials and other stakeholders to, at least in part, ensure that resilience planning is well catered for in the SDFs. As noted by Van Aswegen (2018), although the skills training aspect is often not given appropriate attention, the training of stakeholders is important for the successful formulation and implementation of regional policy. Community representatives and stakeholders operating in different sectors should be accommodated because the SDFs (as components of integrated development planning) are meant to play an integrative role in guiding planning and development in a given area.

In terms of empirical research, it is recommended that future studies go beyond the analysis of regional resilience in the plans (SDFs) in the manner of the current study, and focus on the analysis of the actual resilience of rural regions. It is hoped that the study lays a foundation for such implementation-related analysis. Similarly, analysis should be undertaken on factors that influence the lack of support, capacity, commitment and integration uncovered in the study. This would provide insights on whether the reasons are for instance political or merely the inability of municipalities to formulate and implement appropriate plans for promoting regional resilience.
